# Dual-functional Nanocarrier for Improved Immunochemotherapy of Acute Myeloid Leukemia

**DOI:** 10.21203/rs.3.rs-9901177/v1

**Published:** 2026-06-18

**Authors:** Song Li, Zhuoya Wan, Limei Wu, Yiqing Mu, Bei Zhang, Jing-Zhou Hou, Jingjing Sun, Wei Du

**Affiliations:** University of Pittsburgh School of Pharmacy; University of Pittsburgh School of Pharmacy; University of Pittsburgh School of Medicine; University of Pittsburgh School of Pharmacy; University of Pittsburgh; University of Pittsburgh Cancer Center; University of Nebraska Medical Center; University of Pittsburgh

## Abstract

Advances with the 7 + 3 regimen and liposomal CPX-351 have significantly improved the treatment of acute myeloid leukemia (AML); however, challenges like drug resistance and immune evasion persist. Despite the demonstrated potential of immunotherapy in treating several types of solid tumors, its impact on the treatment of AML remains limited. To address these challenges, we engineered an ultrasmall (~ 15 nm) cytarabine (AraC)-based polymeric micelle nanocarrier (PAraC) for efficient AML cell uptake and bone marrow niche targeting. scRNA-seq and real-world dataset analysis identified upregulated TLR7/8 in malignant AML cells, particularly after 7 + 3 treatment, supporting the inclusion of the TLR7/8 agonist R848 in our combination therapy. Our strategy integrates smart co-delivery of AraC, daunorubicin (Daun), and R848 using the PAraC platform. This triple nano-combination (PAraC/Daun/R848) demonstrated potent antitumor efficacy, outperforming CPX-351 and PAraC/Daun in multiple leukemia models, including murine and patient-derived xenografts (PDX) AML models and AraC-resistant acute lymphoblastic leukemia (ALL) model. Notably, PAraC/Daun/R848 reversed immunosuppressive phenotypes seen with CPX-351 or PAraC/Daun and induced robust leukemia cell maturation. With its modular design, efficient delivery, and remarkable preclinical performance, the PAraC platform holds immense promise for clinical translation, offering a new frontier for immunotherapeutic interventions in AML.

## Introduction

Acute myeloid leukemia (AML), the most common acute leukemia in adults, is a type of blood cancer with marked cytogenetic and molecular heterogeneity, characterized by an arrest of maturation and aggressive proliferation of immature myeloid progenitor cells [1]. It progresses quickly over weeks to months without treatment [1, 2]. This leads to a low average five-year survival rate among all ages of AML, which is about 31% [3]. The standard treatment of AML in young and fit patients over the last 40 + years has been induction therapy with continuous intravenous (i.v.) infusion of cytarabine (AraC) in combination with anthracycline (e.g., daunorubicin (Daun)) (7 + 3 regimen) followed by consolidation with high-dose AraC or allogeneic stem cell transplant depending on the prognostic features [4].

One major issue with the use of free drugs, alone or in combination, for cancer treatment including AML, is their rapid elimination from the blood. In addition, AraC is subjected to rapid inactivation by deaminase [5]. The rapid clearance and metabolism of AraC necessitates a continuous i.v. infusion protocol that requires the patient to be in the hospital and have prolonged IV cannulation. Another issue in combination therapy is the difficulty in the simultaneous co-delivery of different agents to cancer cells. Improvement in drug bioavailability and/or co-delivery of multiple drugs via a nanocarrier represents a promising strategy to improve the outcome of treatment in addition to improving patient compliance [6]. One major advancement in AML therapy is the development of CPX-351, a liposomal co-formulation of AraC and Daun at a 5:1 ratio (m/m), which has clearly demonstrated a therapeutic benefit over the standard combination therapy in patients with secondary and high-risk AML [7]. However, CPX-351 has a complicated manufacturing process, which significantly increases the cost of treatment. In addition, loading another drug into CPX-351 is extremely challenging [1, 8, 9].

Immunotherapy has revolutionized the treatment of both solid and liquid malignancies [10, 11]. However, only the anti-CD33 antibody drug conjugate gemtuzumab ozogamicin is currently approved as an antibody-targeted therapy for AML [12]. AML is challenging to treat as they are biologically very heterogenous with diverse genetic driver mutations and cytogenetic abnormalities [13]. The success of immune checkpoint blockade (ICB) in AML has been modest to date [14]. Combinations of ICB with hypomethylating agents (HMAs) has led to improved efficacy, which is attributed, at least partially, to upregulation of checkpoint molecules [15].

Toll-like receptors (TLR) agonists have been reported to enhance antitumor immunity in several solid tumor models [16]. However, exploration of their immunomodulating activity to improve the treatment of AML has been understudied. Interestingly, several types of TLRs are expressed in cancer cells of AML patients, and treatment with a TLR agonist, particularly a TLR7/8 agonist, led to tumor cell differentiation and apoptosis via a mechanism that is independent of their immunostimulatory activity [17]. R848, a TLR7/TLR8 agonist, considerably impairs the growth of human AML cells and improves AML cell differentiation in immunodeficient mice [18]. However, more research is needed to understand how R848 works in leukemia and to explore the benefits of combining R848 with chemotherapy, such as cytarabine, for leukemia treatment.

In this study, we demonstrated through single-cell RNA-seq analysis and real-world datasets that TLR7/8 are significantly upregulated in AML cells in human patients. Moreover, TLR7/8 expression was further elevated following 7 + 3 treatment cycles. These findings provide compelling evidence to repurpose the TLR7/8 agonist R848 for leukemia treatment and emphasize the therapeutic potential of combining chemotherapy with a TLR7/8 ligand for AML. To address persistent challenges such as drug resistance and immune evasion in AML, we developed a novel cytarabine (AraC)-based polymeric micelle nanocarrier (PAraC) capable of co-delivering AraC and daunorubicin (Daun). This innovative triple nano-combination (PAraC/Daun/R848) was thoroughly characterized for its biophysical properties and extensively evaluated for in vitro and in vivo therapeutic efficacy across various leukemia models, including AML PDX model and AraC-resistant acute lymphoblastic leukemia (ALL) model. Mechanistic studies revealed that the triple combination facilitated efficient cellular uptake by AML cells and precisely targeted bone marrow niches where leukemia cells reside. Notably, PAraC/Daun/R848 reversed immunosuppressive phenotypes observed with CPX-351 or PAraC/Daun, while inducing robust leukemia cell maturation—a critical milestone toward therapeutic success.

## Results

### TLR7/8 expression is upregulated after chemotherapy in AML patients.

**Figure 1:**
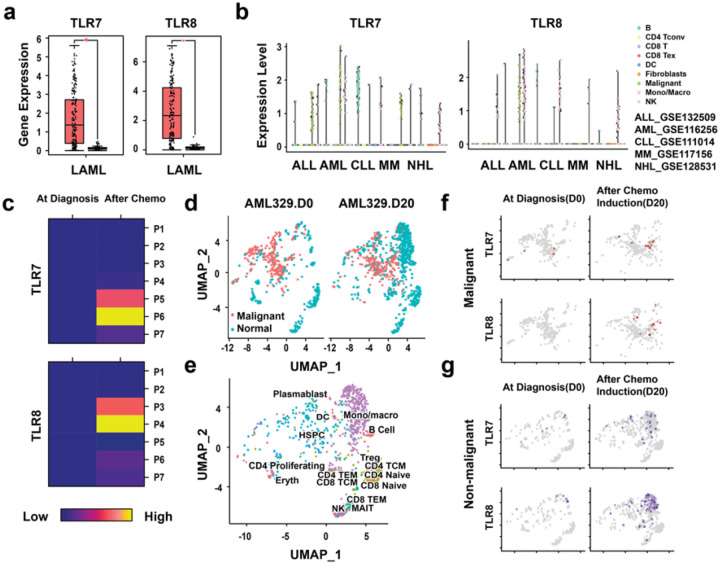
Bioinformatics analysis of TLR7/8 expression in the bone marrow (BM) of AML patients. (**a**) Expression levels of TLR7/8 in the BM of AML patients and healthy controls. (**b**) Expression levels of TLR7/8 in the BM of different subpopulations of malignant cells and immune cells from patients with acute lymphoblastic leukemia (ALL), adult acute myeloid leukemia (AML), chronic lymphocytic leukemia (CLL), multiple myeloma (MM), and non-Hodgkin lymphoma (NHL). (**c-g**) Changes in the expression levels of TLR7/8 in BM (**c**), malignant cells (**f**) as clustered in (**d**), and some immune cells subpopulation (**g**) as clustered in (**d, e**).

The TLR7/TLR8 agonist R848 has been reported to have an immunostimulatory effect in both leukemia and other solid tumors in a TLR7/TLR8-dependent manner [19, 20]. TLR7/8 have been shown to be expressed in AML cells [21, 22]. To further study the expression of TLR7/8 in patients with different types of hematological malignancies before and after chemotherapy, we analyzed publicly available single-cell (sc) RNA-seq data of patients' bone marrow (BM) aspirates [23]. As shown in Fig.1a, the expression levels of both TLR7 and TLR8 were significantly upregulated in AML patients compared to healthy individuals. The increased expression of TLR7/8 was also seen in patients diagnosed with ALL, Chronic myeloid leukemia (CML), multiple myeloma (MM), and non-Hodgkin lymphoma (NHL) (Fig. 1b). Further analysis indicates that the overexpressed TLR7/8 are mainly distributed in leukemia cells and leukemia-associated monocytes/macrophages (Fig. 1b). Interestingly, TLR7/8 expression was further increased following AraC/Daunorubicin induction therapy in the bone marrow of some AML patients despite some variations among patients (Fig. 1c). Further analysis of all cell populations (Fig. 1d–e) shows that TLR7/8 expression remains concentrated to leukemia cells (Fig. 1f) and monocytes/macrophages (Fig. 1g) after chemotherapy. These data suggest the potential synergistic effect of combining R848 with the “7+3” regimen in AML treatment.

### Preparation of ultrasmall nanocarriers for triple combination therapy

**Figure 2:**
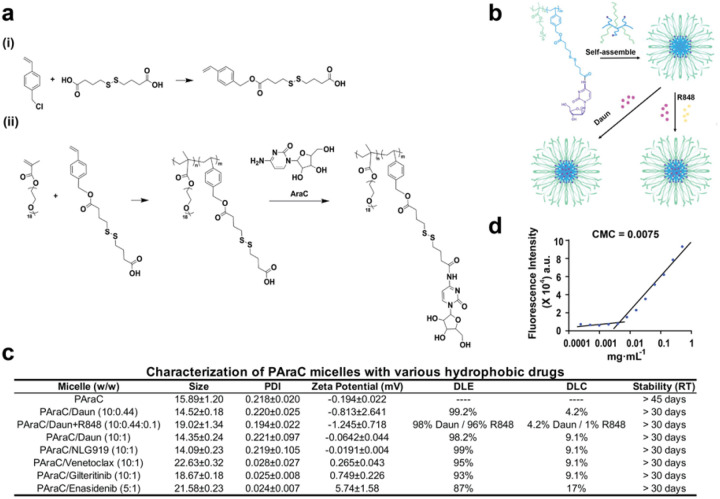
Synthesis and characterizations of PAraC and drug loaded PAraC NPs. (**a**) Synthesis route of the PAraC polymer through RAFT polymerization followed by the conjugation of AraC. (**b**) PAraC polymer and hydrophobic drug can self-assemble into nanoparticles in water after film hydration. (**c**) Both empty and drug-loaded PAraC nanoparticles showed ultra-small particle sizes and nearly neutral zeta potential (N=3). All drug-loaded formulations achieved around 90% drug loading efficiency and stability at room temperature for more than 30 days. (**d**) Critical micelle concentration (CMC) was measured by the fluorescence intensity of the loaded fluorescence probe at different PAraC concentrations.

AraC has the issue of rapid clearance and metabolism. To increase its t_1/2_ in blood, we developed an AraC prodrug (PAraC) via conjugation with POEG-co-PVD (PVD) polymer. Such modification shall also protect its degradation by deaminase. PAraC was synthesized through reversible addition−fragmentation chain-transfer (RAFT) polymerization followed by conjugation of AraC to the polymer backbone (Fig. 2a). The polymer structures were characterized by ^1^H-NMR (**Supplementary Fig. 1**). Both PVD and PAraC are amphiphilic polymers and could self-assemble to form micelle nanoparticles (NPs). PVD NPs were ~160 nm in size and showed limited drug loading capacity in co-formulating Daun and R848. Interestingly, conjugation of AraC to POEG-co-PVD led to a drastic reduction of the particle size from 160 to ~15 nm. In addition, the resulting PAraC ultrasmall NPs demonstrated significantly improved performance in co-loading Daun and R848. Fig. 2b–c show the biophysical properties of PAraC NPs loaded or co-loaded with various hydrophobic drugs including Daun, R848, NLG919 etc. In particular, PAraC NPs co-loaded with Daun and R848 were stable and remained small in size (~20 nm) at RT for over one month. In addition, PAraC NPs have a low critical micelle concentration (CMC) of 0.0075 mg/mL (Fig. 2d), suggesting the excellent colloidal stability of PAraC NPs after dilution in blood following systemic administration.

### In vitro and in vivo cellular uptake of PAraC NPs

**Figure 3:**
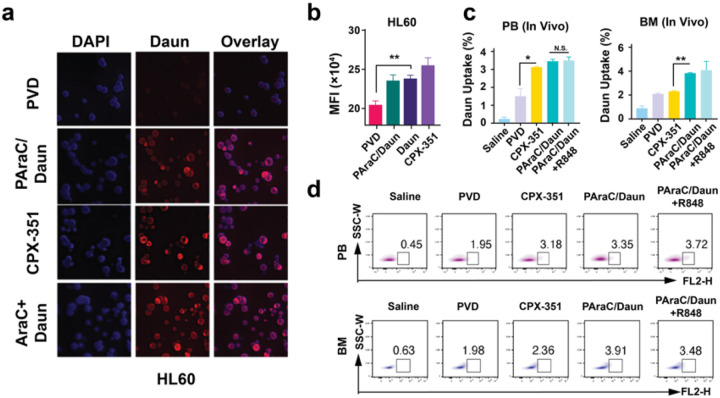
In vitro and in vivo uptake of PAraC NPs. The HL60 cell line was treated with free daunorubicin or nanoparticles loaded with daunorubicin for 4 hours. After treatment, cell samples were analyzed by either fluorescence imaging (**a**) or flow cytometry **(b**) to measure the cellular uptake of different formulations (N=3). (**c**-**d**) Different daunorubicin-loaded nanoparticles were i.v. injected into mice (N=5). PB and BM cells were later isolated for flow cytometry.

The cellular uptake of NPs could be affected by their sizes [24, 25]. We hypothesized that the ultra-small-sized PAraC NPs may have improved cellular uptake compared to PVD NPs (~160 nm). We first tested the cellular uptake of different formulations in vitro. Daun was either loaded in NPs or used as a free drug to track the cellular uptake. The amount of Daun taken up by cells was measured by the fluorescence intensity of Daun. Fluorescence microscopy showed that the small-sized PAraC NPs were more effectively taken up by the cultured HL-60 cells compared to the large-sized PVD NPs (Fig. 3a). Quantitative analysis by flow cytometry showed similar results (Fig. 3b). CPX-351 showed slightly better cell uptake than PAraC NPs, as shown by flow cytometry (Fig. 3b), likely due to the lipophilic nature of liposome NPs.

Compared to in vitro cellular uptake, systemically administered NPs undergo a far more complex process to reach target cells. Using a MLL-AF9 knock-in murine AML model [26] that well replicates human AML genetics and supports robust studies of leukemogenesis and drug responses, we evaluated the in vivo cellular uptake of various NPs following intravenous administration. Peripheral blood and bone marrow samples were analyzed via flow cytometry. Consistent with in vitro findings, PAraC NPs exhibited higher uptake than PVD NPs in both peripheral blood and bone marrow (Fig. 3c–d). While PAraC and CPX-351 NPs demonstrated comparable uptake in peripheral blood, PAraC NPs showed significantly greater uptake in bone marrow (Fig. 3c–d).

### Pharmacokinetics (PK) and biodistribution of PAraC NPs

A NIR fluorescence dye, DiR, was used to examine the PK of the drug loaded in PAraC [27]. DiR in free form or formulated into PAraC NPs was injected into mice. Blood was collected at different time points, and the concentration of DiR was measured by fluorescence intensity. As shown in **Supplementary Fig. 2a**, free DiR was rapidly cleared from the blood, while the formulated dye stayed in the blood for a prolonged period. Twenty-four hours after injection of free DiR or DiR-loaded NPs, mice were sacrificed, and hind limb bones were collected. Accumulation of DiR in bone marrow was measured by fluorescence imaging (**Supplementary Fig. 2b**) and quantified by the fluorescence intensity (**Supplementary Fig. 2c**). Free DiR showed little accumulation in bone marrow, as indicated by the dim signals from the hind limb bones (**Supplementary Fig. 2b**). Incorporation of DiR into PVD or PAraC NPs led to significant increases in the signals of DiR in the bones. The amounts of DiR signals in PAraC group were significantly higher than those in mice treated with DiR-loaded PVD NPs (**Supplementary Fig. 2b-c**). The enhanced bone targeting of PAraC NPs is likely attributed to the effective cellular uptake of these NPs in bone marrow as shown in Fig. 3c–d. This is highly significant as bone marrow represents a major target tissue where leukemia cells especially leukemia stem cells, reside and proliferate, contributing to the treatment resistance and relapse.

### In vivo antitumor effect of PAraC NPs in C1498 model

The therapeutic efficacy of PAraC NPs was first tested in murine AML models. C1498 is an aggressive murine AML cell line that is highly metastatic and highly lethal [28, 29]. The C1498 model was established by i.v. injection of C1498 cells into C57BL/6J mice [30]. As shown in **Supplementary Fig. 3a**, engraftment was conducted on Day 0. On Day 4, different treatments were given i.v. to the mice every two days for five times and the survival of the mice was monitored. **Supplementary Fig. 3b** shows that PAraC alone or the free AraC/Daun/R848 combination showed a modest effect in prolonging the survival of C1498 leukemia cells-engrafted mice. Incorporation of Daun into PAraC NPs led to an improvement in survival (**Supplementary Fig. 3b**). Survival was further improved in mice treated with PAraC NPs co-loaded with Daun/R848 (**Supplementary Fig. 3b**). To study the immune profile in bone marrow after different treatments, mice were sacrificed, and bone marrow was collected for flow cytometry on Day 22. **Supplementary Fig. 3c** shows that treatment with the free drug combination led to significant decreases in the numbers of CD4^+^ T cells, CD8^+^ T cells, and NK cells in the bone marrow. In contrast, co-delivery of AraC, Daun, and R848 via PAraC NPs led to significant increases in the numbers of both total and activated (CD69^+^) CD4^+^ T cells, CD8^+^ T cells, and NK cells in bone marrow (**Supplementary Fig. 3c**). The numbers of these immune cell subsets in the PAraC/Daun/R848-treated group were also higher than those in the saline-treated group, suggesting that our approach can not only boost the tumor immune microenvironment but also overcome the immune suppression associated with the free drug combination.

### In vivo antitumor effect of PAraC NPs in MLL-AF9 model

We then examined the therapeutic efficacy of PAraC NPs in another mouse AML model that showed more clinical relevance to human AML [31]. In this model, the expression of the oncogenic MLL-AF9 fusion protein results in the development of AML beginning around 5 months of age [31]. Rearrangements of the mixed lineage leukemia (MLL) gene cause aggressive ALL and AML that generally respond poorly to treatment [32]. The MLL-AF9 knock-in mouse model reflects aspects of AML in humans and can be used to provide biological insights into MLL-rearranged leukemogenesis and assess the therapeutic efficacy of novel therapies for patients with this genotype [32].

**Figure 4:**
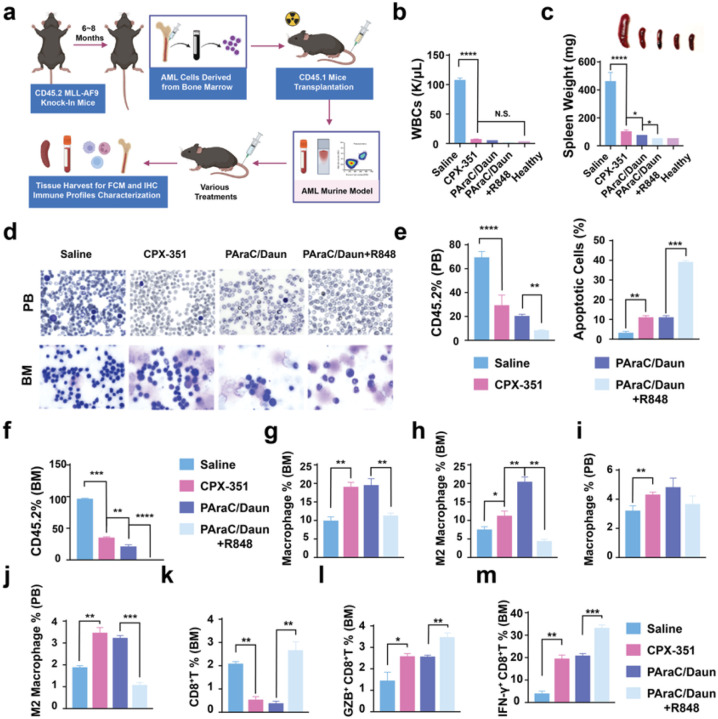
In vivo therapeutic efficacy of PAraC NPs. (**a**) Workflow of MLL-AF9 tumor model establishment, treatment, and sample testing (created in BioRender). Multiple indicators of acute myeloid leukemia progression were measured in different groups after treatments, including white blood cell counts (WBC) (**b**), spleen weights (**c**), and percentages of remaining leukemia cells (in PB and BM) and peripheral blood apoptotic cells (**d-f**). Macrophage infiltration and M2 macrophage polarization in BM (**g-h**) and PB (**i-j**) were also tested in different treatment groups. (**k-m**) T cell infiltration in BM was tested in each group (N=5).

Fig. 4a illustrates the tumor model establishment and treatment timeline. At 6 to 8 months of age, MLL-AF9 knock-in C57BL/6J mice (CD45.2^+^) spontaneously developed leukemia. Leukemia cells were harvested from the bone marrow and transplanted into B6 recipient mice (CD45.1^+^) preconditioned with sublethal irradiation. Leukemia diagnosis was confirmed through white blood cell (WBC) counts, Giemsa staining, and flow cytometry. Thirty days after AML cell engraftment and leukemia diagnosis, different treatments were initiated. Five days after completing three treatment cycles, mice underwent various examinations. The engraftment of CD45.2^+^ AML cells in CD45.1^+^ mice and disease progression were readily assessed using CD45.2 antibody staining and flow cytometry.

PAraC/Daun was more effective than CPX-351 in controlling the leukemia burden as shown by lower numbers of leukemia cells in blood and BM and reduced spleen weights. PAraC NPs coloaded with Daun+R848 showed the superior antitumor activity with almost normalized spleen weights (Fig. 4b–f). Interestingly, treatment with CPX-351 or PAraC/Daun led to increased infiltration of macrophages in BM and polarization to M2 phenotype, which is known to be pro-tumor [33]. These changes were completely reversed in mice treated with the triple combination therapy (Fig. 4g–j). Fig. 4k–m show that treatment with CPX-351 or PAraC/Daun resulted in increases in the numbers of functional (GZB^+^) CD8^+^ T cells; however, both treatments were associated with significant decreases in the total numbers of CD8^+^ T cells. No decreases in CD8^+^ T cells were seen in the triple combination group. In addition, more GZB^+^ CD8^+^ T cells were seen in this group compared to mice treated with CPX-351 or PAraC/Daun.

### In vivo antitumor effect of PAraC NPs in a PDX model

**Figure 5:**
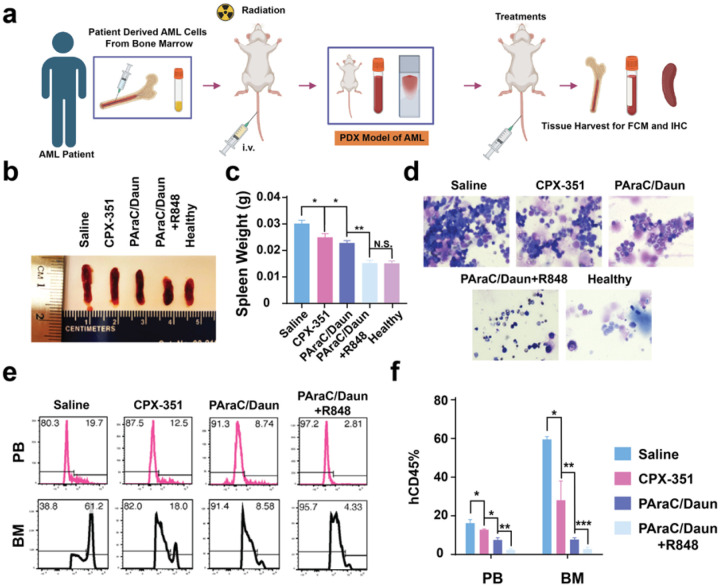
In vivo antitumor effect of PAraC NPs in the PDX model. (**a**) Workflow of PDX model establishment, treatment, and sample testing (created in BioRender). (**b-c**) Sizes and weights of spleen from mice inoculated with AML cells and undergoing different treatments (N=5). Naïve healthy mice were used as a control. (**d**) Giemsa staining of bone marrow from mice inoculated with AML cells and undergoing different treatments. (**e-f**) After different treatments, blood and bone marrow were isolated from mice, and the hCD45^+^ numbers were measured and quantified by flow cytometry (N=5).

To further evaluate the therapeutic efficacy of our NPs in a clinically relevant setting, we established a PDX leukemia model using NSGS mice, as illustrated in Fig. 5a. Bone marrow cells from a UPMC patient were transplanted into NSGS mice preconditioned with sublethal irradiation. NSGS mice were selected due to their severely immunodeficient background and transgenic expression of human cytokines (IL-3, GM-CSF, and SCF), which facilitate the engraftment and proliferation of human leukemia cells, thereby improving model reliability and more accurately mimicking the human leukemic microenvironment. Leukemia diagnosis was confirmed by WBC counts and Giemsa staining. Treatments were initiated 30 days after AML cell transplantation and administered every three days for a total of three doses. Mice were sacrificed 10 days following the final treatment and subjected to comprehensive assessments. Disease progression was evaluated using human CD45 (hCD45) antibody staining, flow cytometry, and immunohistochemistry (IHC).

Inoculation of AML cells led to significant increases in both the sizes and weights of mouse spleens without treatment (Fig. 5b). The spleen sizes and weights were significantly decreased following treatment with CPX-351 or PAraC/Daun but remained to be significantly raised compared to normal spleen. On the other hand, PAraC/Daun/R848 treatment decreased the spleen volumes and weights to a normal range (Fig. 5b–c).

Bone marrow smear shows significantly increased leukemic blasts in the group receiving no treatment. Treatment with CPX-351 or PAraC/Daun led to decreased leukemic blasts; however, the number of leukemia blast remained more dramatic compared to the bone marrow from normal mice. Incorporation of R848 into PAraC/Daun led to a further decrease in leukemia blast frequencies (Fig. 5d). In addition, decreases in the numbers of hCD45^+^ AML cells were seen in both peripheral blood and BM in all three treatment groups, with PAraC/Daun/R848 group showing the lowest number of hCD45^+^ AML cells (Fig. 5e–f).

### In vivo antitumor effect of PAraC NPs in ALL models

In addition to AML models, we also tested the treatment effect in acute lymphoblastic leukemia (ALL) models. A murine ALL model was first established by i.v. inoculation of luciferase (Luc)-expressing p185^+^ BCR-ABL^+^Arf^−/−^ pre-B cells into C57Bl/6 mice at Day 0. Ten days after inoculation, different treatments were given to the mice (every 3 days for a total of 3 treatments). Leukemia progression was monitored by bioluminescence imaging. As seen in **Supplementary Fig. 4a**, all mice in the saline group died after day 19, and all mice in the PAraC group died after Day 25. The disease progressed rapidly in the PAraC/Daun group after day 19 and only one mouse remained alive on day 31. In consistent with the data in AML models, PAraC/Daun/R848 demonstrated superior antitumor activity. ALL was well controlled and, after day 19, no signals of ALL luminescence were detected up to day 31, the last day followed in this experiment.

**Figure 6:**
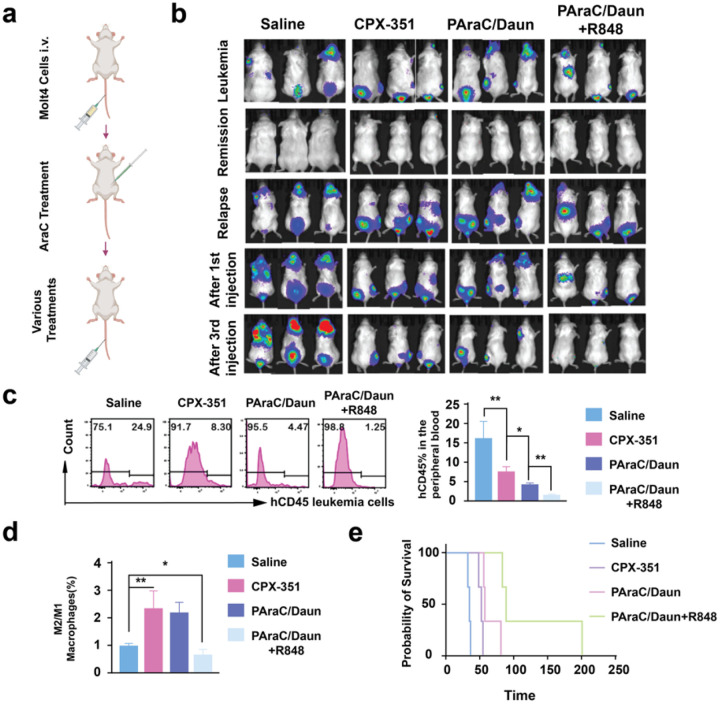
In vivo antitumor effect of PAraC NPs in human AraC resistant ALL models. (**a**) Workflow of human AraC-resistant ALL model establishment (created in BioRender). (**b**) The model was established by engrafting mice with Molt4-Luc2 cells. Establishment and progress of the ALL after different treatments were monitored by bioluminescence imaging on different days (N=3). (**c-d**) Submandibular blood was collected for flow analysis. hCD45^+^ cell number was measured as an indicator for leukemia progress (**c**). M1/M2 ratios were measured to study the immune profile after different treatments (**d**). (**e**) Survival curves of the mice from different treatment groups.

A human AraC-resistant ALL model was also established to study the efficacy of PAraC NPs [34] (Fig. 6a). Human Molt4-Luc ALL cells were intravenously injected into NSGS mice and leukemia establishment was monitored using whole-body imaging. Mice were then treated with 50 mg/kg of free AraC for a total of five doses until no detectable signal was observed in the NSGS mice. The mice were then monitored for reappearance of Luc signals, an indication of relapse from residual AraC-resistant ALL cells (Fig. 6a–b). Mice were treated again with different PAraC formulations. In addition to monitoring tumor progression via whole-body imaging (Fig. 6b), small amounts of blood were collected from the*cheek*(*submandibular*) and subjected to flow analysis of hCD45^+^ leukemia cells and M1/M2 ratios on day 30 (Fig. 6c–d). The mice were then followed for survival (Fig. 6e). Similar to what was shown in other models, PAraC/Daun/R848 showed the highest therapeutic efficiency, followed by PAraC/Daun and CPX-351. We similarly observed increased macrophages in peripheral blood and polarization towards the M2 phenotype in NSGS mice following treatment with CPX-351 or PAraC/Daun (Fig. 6d). Again, these changes were completely abolished in the group with triple combination treatment.

### Safety profile of drug loaded PAraC NPs

**Figure 7:**
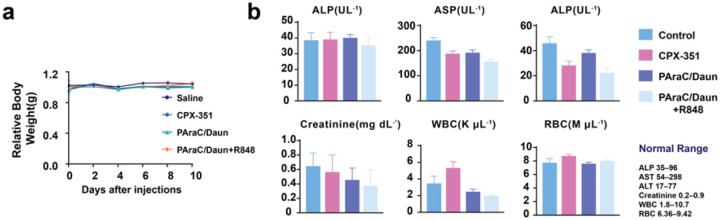
Safety profiles of Daunorubicin/R848 co-loaded PAraC nanoparticles (NPs). **(a)** Changes in mouse body weights over the course of treatments. (**b**) Serum levels of alkaline phosphatase (ALP), aspartate aminotransferase (AST), alanine aminotransferase (ALT), and creatinine, as well as white blood cell (WBC) and red blood cell (RBC) counts, following the indicated treatments.

A preliminary study was conducted to assess the toxicity profile of PAraC/Daun and PAraC/Daun+R848 in naïve mice with CPX-351 as a control. As shown in Fig. 7a–b, repeated dosing of either formulation was associated with minimal toxicity at the doses used as evident from normal ranges of a number of parameters examined including body weights, blood chemistry, and blood cell counts.

### Induction of maturation in AML cells by different treatments

**Figure 8:**
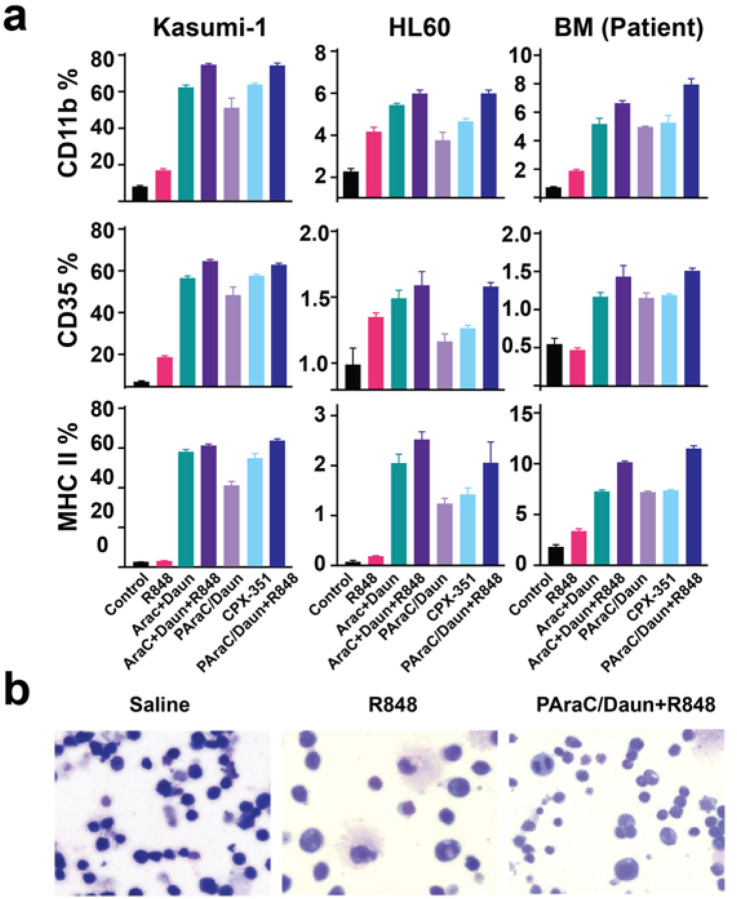
Induction of AML cell maturation following different treatments. Kasumi-1 cells, HL60 cells, and patient-derived bone marrow cells were treated as indicated. Expression of the monocyte maturation markers CD11b, CD35, and MHC II was evaluated following treatments (**a**). Morphological changes in Kasumi-1 cells were further assessed by microscopic examination after Giemsa staining (**b**). N = 5.

Treatments with either AraC/Daun [35, 36] or R848 [18, 37] have been reported to have a positive effect on leukemic cell maturation. Thus, we also investigated the induction of leukemic cell maturation by different NP treatments. Fig. 8a shows the expression of several monocyte maturation markers following different treatments of either human leukemia cell lines or patient bone marrow (BM) aspirates. AraC/Daun treatment led to increased expression of several monocyte maturation markers, including CD11b, CD35, and MHCII. R848 treatment similarly induced the expression of these markers. Importantly, the triple combination, either as a free drug combination or as co-loaded NPs, led to the highest expression levels of the three markers examined. In consistent with changes in the expression of maturation markers, a more differentiated cell morphology was observed for Kasumi-1 cells after treatment with PAraC/Daun/R848 (Fig. 8b).

### Upregulation of PD-1 expression in CD8^+^ T cells after PAraC/Daun/R848 treatment

Combination therapies of anti-PD-1 with chemotherapy have shown enhanced anti-tumor efficacies [38, 39]. The anti-PD-1 response is associated with PD-1 expression in CD8^+^ T cells in some cancers [40]. To investigate the potential of combination therapy of anti-PD-1 and our NPs, we tested the PD-1 expression in CD8^+^ T cells after different treatments. **Supplementary Fig. 5** showed that the enhanced therapeutic effect of PAraC/Daun+R848 was associated with upregulation of PD-1 in CD8^+^ T cells among different treatments. This indicates that combination of PAraC/Daun+R848 with anti-PD-1 is likely to lead to a further improvement in antitumor activity.

## Discussion

We reported in this study an ultrasmall, dual-functional nanocarrier that is highly effective in codelivery of AraC, Daun, and R848 and demonstrated significantly enhanced therapeutic efficacy and improved tumor immune microenvironment in several murine and human AML and ALL models including PDX model.

Small-sized nanocarriers have gained increasing attention as a strategy for targeting of solid tumors, which not only enhances tumor accumulation but also facilitates tumor penetration, particularly in tumors enriched with dense stroma such as pancreatic cancer and breast cancer [41]. Interestingly, our data also demonstrated significant advantages of small-sized NPs in targeting liquid tumors compared to the large-sized counterparts. In addition to more effective cellular uptake by AML cells compared to large-sized PVD NPs, PAraC were more effective in targeting BM. This is highly significant as BM represents a major target tissue where leukemia cells especially leukemia stem cells reside and proliferate, contributing to the treatment resistance and relapse.

Cellular uptake of NPs has been shown to be affected by the size and geometry in addition to surface charge [42]. Small NPs have been shown to be more effective in cellular uptake than large NPs [43]. However, the detailed mechanism for the uptake of PAraC NPs by AML cells remains largely unknown. Recently serum proteins that are recruited to the surface of NPs, also known as protein corona, have been shown to significantly affect the tissue homing and subsequently the cellular uptake of NPs [44]. The surface properties of the NPs significantly affect the amounts and protein compositions in protein corona [45]. We have recently reported that ultrasmall-sized NPs based on azacitidine prodrug (PAZA) selectively recruit fibronectin from serum to the surface, which contributes significantly to the effective targeting of PAZA NPs to solid tumors [46]. It remains to be investigated whether fibronectin or other serum proteins also plays a role in the increased cellular uptake of PAraC NPs by AML cells. It should be noted that, although leukemia is considered a “liquid tumor” characterized by dissemination of BM-derived cells in the blood, it can sometimes spread to other parts of the body, such as the lymph nodes, spleen, liver, brain, skin, and gums [47, 48]. Occasionally, AML cells can form solid tumor masses referred to as myeloid sarcoma or chloroma that can develop anywhere in the body [49, 50]. Therefore, ultrasmall NPs such as PAraC may have an additional advantage of targeting these rare lesions due to their unique physical property.

There are several other advantages with PAraC-based nanocarrier over other systems including CPX-351. PAraC-based system is simple, and the PAraC NPs loaded or co-loaded with Daun and R848 can be readily prepared via a simple self-assembling protocol. Considering AraC as a backbone therapy for AML and other malignancies, various new combination therapies can be readily developed via incorporating newly developed therapeutic agents into PAraC-based NPs. On the other hand, development of CPX-351 involves a complicated protocol and it is extremely challenging to incorporate another new agent into the current CPX-351 formulation. As an AraC prodrug, PAraC also offers protection of AraC against deaminase-mediated degradation and provides sustained action over a relatively long period of time. It should also be noted that ultrasmall NPs can be similarly developed with other nucleotide analogues such as azacitidine and decitabine that are also major drugs for leukemia treatments. Therefore, our technology represents a platform that may be used for the development of diverse types of combination therapies that are based on ultrasmall NPs-mediated codelivery.

PAraC/Daun was more effective than CPX-351 in several murine and human AML/ALL models including PDX model, and addition of R848 led to further improvement in the therapeutic efficacy of PAraC/Daun. It was noted both CPX-351 and PAraC/Daun caused an immunosuppressive tumor microenvironment as shown by decreased numbers of CD8^+^ T cells and polarization of macrophages towards a M2 phenotype. Importantly, these changes were completely reversed in the PAraC/Duan/R848 triple therapy group. TLR7-based immunotherapy has been shown to improve TIME in solid tumor models through decreasing the numbers of immunosuppressive immune cells such as MDSCs and blocking their functions [51]. More studies are needed to further understand how R848 improves TIME in AML and ALL models.

Induction of cell maturation represents an important mechanism for various AML therapies including AraC/Daun-based therapy [52]. R848 has also been shown to induce AML cell maturation in a TLR7/8-dependent manner [18, 53]. Importantly, combination of the 3 drugs either as free drugs or co-loaded NPs led to further enhancement of AML cell maturation. In addition to direct impact on leukemia cells, cell maturation shall facilitate the antigen presentation and contribute to the overall immune response [54, 55]. It is interesting to note that the expression levels of TLR7/8 are upregulated in AML cells in patients after chemotherapy. Therefore PAraC/Daun/R848 might be particularly suitable for AML patients who fail or become resistant to AraC/Daun-based treatments. In a preliminary study with an AraC-resistant ALL model, we showed that PAraC/Daun/R848 was much more effective than either CPX-351 or PAraC/Daun in controlling the disease progression. More studies are needed to further understand the underlying mechanism.

Anti-PD-1/PD-L1 has become a promising immunotherapy for certain types of solid tumors. However, anti-PD-1/PD-L1 has shown limited efficacy in AML due to, among others, the poorly systemic immune response and several mechanisms of hijacking the immune system via the immune checkpoints [56, 57]. However, the combination of hypomethylating agents and PD-1/PD-L1 inhibitors has shown promising results in acute AML patients, likely due to improvement of TIME [15, 58]. In addition to significant improvement in TIME, PAraC/Daun/R848 triple therapy led to significant upregulation of PD-1 in CD8^+^ T cells, suggesting the potential of combining with anti-PD-1 treatment to further improve the therapeutic efficacy for AML.

There are several reports of nanomedicines for improved AML treatment using other nanocarriers and/or drug combinations. Most of the studies use very few murine or human AML models. Lack of comparison with standard of care is another limitation with some of the studies. In this study, we comprehensively evaluated PAraC/Daun/R848 in several AML models and clearly demonstrated its superiority over CPX-351 in therapeutic outcome, especially in improving the TIME. More studies direct comparison among different nanomedicines may improve our understanding of advantages and disadvantages of each system, which may lead to further improved therapy for AML.

In summary, we have developed a simple and highly effective immunochemotherapy that is based on PAraC NPs-mediated codelivery of AraC, Daun, and R848. More studies are warranted to investigate the mechanism of action and therapeutic potential, especially the potential of combining with PD-1/PD-L1 inhibitors.

## Materials and Methods

### Reagents

Dulbecco’s Modified Eagle’s Medium (DMEM), RPMI 1640 Medium, Fetal Bovine Serum (FBS), Penicillin-Streptomycin (PS), trypsin-EDTA solution, 4-vinylbenzyl chloride, 4,4′-dithiodibutyric acid, 2,2-Azobis (isobutyronitrile), 4-cyano-4-(thiobenzoylthio)pentanoic acid, poly(ethylene glycol) methyl ether methacrylate (average Mn 950), were purchased from Sigma-Aldrich (MO, U.S.A). Daunorubicin (HY-13062A), AraC (HY-13605), R848 (HY-13740) were purchased from MedChemExpress LLC (NJ, USA).

### Cells and Animals

C57BL/6 mice (4–6 weeks) were purchased from Jackson Laboratory (Bar Harbor, ME). MLL-AF9 knock-in mice were obtained from JAX Laboratory (Stock# 009079, Bar Harbor, ME). Humanized NSGS mice, expressing transgenic cDNA encoding human SCF, GM-CSF, and IL-3 (NOD SCID IL-2Rγ−/− SCF, GM-CSF, and IL-3), were obtained from Jackson Laboratories (Stock #013062; Jackson Laboratory, Bar Harbor, ME). Mice of both sexes, aged 8–10 weeks, were used. HL60, Kasumi-1, C1498 were purchased from ATCC (Manassas, VA). Human ALL cell line Molt4-Luc2 expressing luciferase was purchased from America Type Culture Collection (ATCC; Manassas, VA) and cultured in RPMI1640 medium supplemented with 10% FBS [59]. AraC (Cytosine β-D-arabinofuranoside; C1768, Sigma-Aldrich, St Louis, MO) was administered at a concentration of 50 mg/kg for in vivo experiments and at 2 μM for in vitro experiments.

### Animal models and therapeutic efficacy study

Multiple AML and ALL animal models were used in this study as described in the Results section. The establishment of AML and ALL models in immunodeficient mice involved obtaining primary human AML and ALL cells and (human bone marrow) CD34^+^ cells from UPMC Hillman Cancer Center following informed consent. The AML and ALL cell line, primary AML and ALL samples, or hCB CD34 + cells were transplanted into 8–12 week-old humanized NSGS mice through intravenous injection after total body irradiation [59]. Mice received sublethal irradiation at a 2.5 Gy dose 24 hours before transplantation to condition them for engraftment. Bone marrow samples were then aspirated from a long bone at various times under isoflurane anesthesia to assess engraftment by flow cytometry. Following engraftment, the mice were divided into different groups for drug treatment.

The progression of diseases after treatment was monitored by bioluminescence imaging and/or survival of the mice. Small amounts of blood were taken during the treatment at various time points for blood tests. Blood and bone marrow samples were also collected after the treatment was finished and the mice were sacrificed. Parameters such as white blood cell count, spleen weight, CD45^+^ cell numbers, and apoptotic cell numbers were measured. These features, as indicators of leukemia disease, were used to assess treatment efficacy.

All animals were housed under pathogen-free conditions according to AAALAC (Association for Assessment and Accreditation of Laboratory Animal Care) guidelines. All animal-related experiments were performed in full compliance with institutional guidelines and approved by the Animal Use and Care Administrative Advisory Committee at the University of Pittsburgh.

### PAraC polymer synthesis

PAraC polymer was synthesized through a controlled reversible addition-fragmentation chain transfer (RAFT) polymerization and subsequent AraC conjugation. Briefly, VD monomer was obtained by esterification of vinylbenzyl chloride and 4,4’-Dithiodibutyric acid according to the method we previously reported [60, 61]. Then, AIBN (1.8 mg, 0.0112 mmol), 4-Cyano-4-(thiobenzoylthio)pentanoic acid (8 mg, 0.0287 mmol), poly(ethylene glycol) methyl ether methacrylate (Mn = 950) monomer (762 mg, 0.8 mmol), VD monomer (680 mg, 2.15 mmol) and 2 mL of tetrahydrofuran were added into a Schlenk tube. After three freeze-pump-thawing cycles, the reaction mixture was stirred at 80°C under the protection of N_2_. POEG-co-PVD was obtained by precipitation in ether after polymerization. Finally, POEG-co-PVD polymer (120 mg, 0.17 mmol -COOH), EDC (400 mg, 2.08 mmol), HOBT (160 mg, 1.2 mmol) and DIPEA (400 μL) were dissolved in DMSO (25 mL). Then, AraC (160 mg, 0.66 mmol) was added to the solution and stirred at room temperature for 72 h. The final product PAraC was achieved after dialysis and lyophilization.

### PAraC NPs Preparation

PAraC NPs were prepared by the film hydration method. A certain ratio of PAraC and the drug to be loaded were dissolved in dichloromethane (DCM) and mixed in glass tubes. A film was formed when DCM evaporated. PBS was then added to the tube to resuspend the nanoparticles. Drug-loaded PAraC NPs were formed quickly after PBS was added. Filtration was applied if precipitates appeared after resuspension.

### PAraC NPs Characterization

The critical micelle concentration (CMC) of PAraC NPs was measured using the fluorescent probe 1,6-Diphenyl-1,3,5-hexatriene (DPH). DPH-loaded PAraC NPs were made with a fixed DPH and PAraC ratio. The DPH-loaded PAraC NPs were then diluted to different concentrations, and the fluorescence intensity of the diluted NPs was measured. The concentration at which fluorescence intensity starts to surge proportionally to PAraC concentration is defined as the critical micelle concentration.

The particle sizes and zeta potentials of PAraC NPs were measured by dynamic light scattering (DLS).

To measure the drug loading efficiency (DLE) and drug loading capacity (DLC), drug-loaded NPs were first lyophilized to remove water. Lyophilized NPs were then dissolved in methanol, and the drug amount was measured by high-performance liquid chromatography (HPLC). DLE and DLC were calculated using the formulas below:

DLE = Weight of drug loaded into NPs / Weight of drug added to the formulation

DLC = Weight of drug loaded into NPs / Weight of NPs

The stability of nanoparticles was tracked by monitoring physical properties, such as particle size and zeta potential, after long-term storage at room temperature. The time during which the nanoparticles remain at their ultra-small sizes is recorded to define their stability.

### Cellular uptake study

Daunorubicin-loaded PAraC or PVD NPs were prepared through the method described above. CPX-351 was prepared as reported. For the in vitro cellular uptake study, different daunorubicin-loaded NPs and free daunorubicin were incubated with HL60 cells respectively for 6 hours. After 6 hours, cells were washed with cold PBS three times and fixed with 10% formalin for 10 minutes on ice. Then, 10% formalin was removed, and the samples were again washed with cold PBS three times. For fluorescence imaging, the samples were further stained with DAPI dye and observed under fluorescence microscopy. For flow cytometry, samples were resuspended in HBSS medium containing 1% FBS and then analyzed by a flow cytometry machine.

For the in vivo cellular uptake study, different samples were injected into MLL-AF9 knock-in mice. Six hours after injection, the mice were sacrificed. Cells from peripheral blood and bone marrow were isolated from the mice. The daunorubicin fluorescence intensity of the obtained cells was measured by flow cytometry as an indicator of cellular uptake of nanoparticles or free drug.

### Pharmacokinetics and accumulation study

DiR-loaded PAraC NPs were prepared through the method described above. DiR-loaded NPs and free DiR were then intravenously (i.v.) injected into C57BL/6 mice, respectively. Blood was collected at different time points after injection (0h, 0.5h, 1h, 2h, 4h, 12h, 24h, 48h). Average DiR fluorescence intensity of plasma was then measured as an indication of DiR concentration in blood circulation. Twenty-four hours after injection, the mice were sacrificed and bones from the hind limbs were dissected. The fluorescence imaging of hind limb bones was taken. The fluorescence intensity of hind limb bones was quantified as an indication of accumulation of DiR in bone marrow.

### Bioluminescence Imaging:

Animals anesthetized with isoflurane were imaged using the Xenogen IVIS imaging system 5–10 minutes after intraperitoneal (i.p.) injection of D-luciferin (Caliper Life Sciences, Waltham, MA) at a dose of 150 mg/kg. Five minutes post-injection, images were captured for 30 seconds to 2 minutes using Living Image 4.7 software (Caliper Life Sciences, Waltham, MA), with views taken from both the ventral and dorsal sides of the mice. Emitted photons from Molt4-Luc2 cells, expressed as flux (photons/s/cm^2^/sr), were quantified and analyzed using the Living Image Pro 2.0 software (Caliper Life Sciences, Waltham, MA) [59].

### Flow cytometry

The donor CD45.2^+^ cell numbers in mouse blood and bone marrow were tested after different treatments by flow cytometry to indicate the progress of leukemia. Cells were isolated from bone marrow, peripheral blood, or spleen to be made into single-cell suspensions. Single cells were first incubated with Zombie NIR (BioLegend, 1:1000) on ice for 30 minutes to stain the dead cells. Samples were then incubated with PerCP anti-mouse CD45.2^+^ antibody (BioLegend, 1:200) on ice for 30 minutes to mark the CD45.2^+^ expressed on the surface of malignant cells. Samples were then fixed and resuspended in Hank's Balanced Salt Solution (HBSS) medium supplemented with 1% FBS for flow analysis.

The immune profile after different treatments was also studied by flow cytometry. Mice were sacrificed after different treatments to collect mouse blood, bone marrow, and spleens. Single-cell suspensions were then prepared. Samples were similarly stained with Zombie NIR on ice for 30 minutes. PerCP anti-mouse CD45 (BioLegend, 1:200), Brilliant Violet 480 anti-mouse CD8 antibody (BD Biosciences, 1:200), PE-Cyanine7 anti-mouse IFN-γ antibody (BD Biosciences, 1:200), eF450 anti-mouse GzmB antibody (eBioscience, 1:200) were used to mark CD8^+^ cells and their subpopulations; APC anti-mouse CD11b antibody (eBioscience, 1:200), Brilliant Ultraviolet 737 anti-mouse CD80 antibody (BD Biosciences, 1:200), and PE anti-mouse CD163 antibody (BioLegend, 1:200) were used to mark macrophages and their subpopulations. Samples were incubated on ice for 30 minutes, then fixed and resuspended in HBSS with 1% FBS for flow analysis.

### Giemsa staining

Blood samples were taken from mice receiving different treatments. Blood films were prepared and left to air dry. The blood films were then fixed with 10% methanol for 10 minutes. After the methanol was air-dried, the blood films were stained with 5% Giemsa stain for 20 minutes and rinsed with water. The obtained films were observed under a microscope to study the leukemia pathology.

### Safety study

PAraC NPs, CPX-351, and saline were given to the mice with a repetitive dose schedule; specifically, injections were given every two days, with three injections in total. Both PAraC NPs and CPX-351 injected into mice contained AraC, Daun, and R848 at doses of 10, 4.4, and 2 mg/kg, respectively. Mouse body weights were monitored during treatment. Five days following the last injection, mouse blood was collected and subjected to different assays, including ALP, AST, ALT, creatinine, and blood cell counts.

## Supplementary Material

This is a list of supplementary files associated with this preprint. Click to download.

- PAraCMSSupplementaryFinal.docx

## Data Availability

The single-cell RNA-sequencing datasets utilized in this study were obtained from the Gene Expression Omnibus (GEO) under the accession number GSE116256. These datasets are publicly available. Further analysis was conducted using the GEPIA (Gene Expression Profiling Interactive Analysis) platform, which provides additional insights from The Cancer Genome Atlas (TCGA) and Genotype-Tissue Expression (GTEx) data.
